# Independent validation of experimental results requires timely and unrestricted access to animal models and reagents

**DOI:** 10.1371/journal.pgen.1008940

**Published:** 2020-06-26

**Authors:** Cassandra R. Diegel, Steven Hann, Ugur M. Ayturk, Jennifer C. W. Hu, Kyung-Eun Lim, Casey J. Droscha, Zachary B. Madaj, Gabrielle E. Foxa, Isaac Izaguirre, VAI Vivarium and Transgenics Core, Alexander G. Robling, Matthew L. Warman, Bart O. Williams

**Affiliations:** 1 Program in Skeletal Disease and Tumor Microenvironment and Center for Cancer and Cell Biology, Van Andel Institute, Grand Rapids, Michigan, United States of America; 2 Orthopedic Research Labs, Boston Children’s Hospital and Department of Genetics, Harvard Medical School, Boston, Massachusetts, United States of America; 3 Musculoskeletal Integrity Program, Hospital for Special Surgery Research Institute, New York, New York, United States of America; 4 Department of Anatomy and Cell Biology, Indiana University School of Medicine, Indianapolis, Indiana, United States of America; 5 Bioinformatics and Biostatistics Core, Van Andel Institute, Grand Rapids, Michigan, United States of America; 6 Vivarium and Transgenics Core, Van Andel Institute, Grand Rapids, Michigan, United States of America; HudsonAlpha Institute for Biotechnology, UNITED STATES

We used CRISPR/Cas9 gene editing to create mice that are lacking *Bglap* and *Bglap2*, which encode osteocalcin [1]. We did not find evidence of increased bone mass, elevated blood glucose levels, or reduced male fertility in our mice [1], which contrasts to what Dr. Karsenty has reported [2–4]. Another group of investigators, working independently of us, created a third *Bglap* and *Bglap2* mouse knockout strain and also failed to substantiate Dr. Karsenty’s results [5]. Furthermore, the osteocalcin-null rat model did not develop obesity, insulin resistance, or glucose intolerance, which conflicts with Dr. Karsenty’s mice [6].

We are pleased that after 24 years Dr. Karsenty has finally made available through JAX the osteocalcin knockout strain he published in 1996. Dr. Karsenty could have donated these mice to JAX, to serve as easy to obtain positive and negative controls for interested investigators, much sooner. Of note, he only submitted these mice to JAX in October 2019, two months after we posted our paper on bioRxiv, and they became available only as cryopreserved stocks the day after our paper was published in *PLOS Genetics*. Specific to the multiple claimed roles of osteocalcin, we urge Dr. Karsenty to also donate his conditional (i.e., floxed) osteocalcin knockout strain since he used that strain as an important independent control in other experiments [4]. These strains along with our knockout mice, which we shipped to JAX on June 17, 2020 after lifting of COVID-19-related shipping restrictions, should enable other independent investigators to study the endogenous role of osteocalcin *in vivo*.

Contrary to what Dr. Karsenty has written, we recognize bone as an endocrine organ as we clearly indicate in our Authors’ Summary [1]. We make no claims regarding whether or not osteocalcin is a hormone. We cannot comment on the protein’s effect when given exogenously, since we did not inject osteocalcin into mice in our study. However, we [1], and others [5–7], found no evidence that supports an endogenous hormonal role for osteocalcin. Should Dr. Karsenty make available batches of his biologically-active osteocalcin without restriction, interested parties could avoid the potential confounder of reagent quality [8] and assess objectively whether osteocalcin has a hormonal role when administered exogenously.

This is not the first time that some of us (CRD, AGR, MLW, and BOW) published data that did not support findings published by Dr. Karsenty. Dr. Karsenty reported that LRP5 controls bone mass by inhibiting serotonin synthesis in the duodenum [9,10]. We found no evidence for this mechanism [11,12]. Of interest, another group studying a larger cohort of patients with the same LRP5 mutation that Dr. Karsenty reported in his original paper [9] could not replicate his findings regarding circulating levels of serotonin [13]. We donated the mice we created for our paper [11] to JAX (Stock numbers 026269, 012668, 012669, 012670, 012671, 012672). The mice created by Dr. Karsenty and used in his experiments still have not been supplied to JAX to our knowledge.

We recognize the importance of fostering integrity in research [14]. This is why we have consistently donated mice we created to JAX for public distribution. We look forward to other investigators using our and Dr. Karsenty’s mice to determine the endogenous role of osteocalcin, meeting the standards of transparency, rigor, and reproducibility upon which the scientific and medical communities rely.
